# Expert consensus on the management of peripheral neuropathy in patients with lymphoma on brentuximab vedotin

**DOI:** 10.1007/s00520-026-11314-5

**Published:** 2026-10-17

**Authors:** Roser Velasco, José A. Sanches, Andreas A. Argyriou, Guido Cavaletti, Eva Domingo-Domenech, Alexander Fosså, Jaime Gállego Pérez-Larraya, Antonia Miranda Ruiz, Alex Molassiotis, André Neder Ramires Abdo, Pablo L. Ortiz-Romero, Susanna B. Park, Julia Scarisbrick, Lauren Urwin, Mian Wang, H. Miles Prince

**Affiliations:** 1 Department of Neurology, Institut Català d’Oncologia–Hospital Universitari de Bellvitge, Bellvitge Biomedical Research Institute (IDIBELL), L’Hospitalet, Barcelona, Spain; 2 Department of Cell Biology, Physiology and Immunology, Centro de Investigación Biomédica en Red Enfermedades Neurodegenerativas (CIBERNED), Universitat Autònoma de Barcelona, Bellaterra, Spain; 3 University of Sao Paulo Medical School, Sao Paulo, Brazil; 4 Agios Andreas General Hospital of Patras, Patras, Greece; 5 Università Di Milano-Bicocca, Monza, Italy; 6 Fondazione IRCCS San Gerardo Dei Tintori, Monza, Italy; 7 Institut Catala d’Oncologia, Hospital Duran Reynals Barcelona, Bellvitge Biomedical Research Institute (IDIBELL), Barcelona, Spain; 8 Oslo University Hospital, Oslo, Norway; 9 Clínica Universidad de Navarra, Instituto de Investigación Sanitaria de Navarra (IDISNA), Pamplona, Spain; 10 University Costa del Sol Hospital Marbella, Málaga, Spain; 11 School of Psychology, Health & Clinical Sciences at, Aston University, Birmingham, UK; 12 University Hospital 12 de Octubre, Institute i+12 Medical School, University Complutense, Madrid, Spain; 13 Faculty of Medicine and Health, The University of Sydney, Sydney, Australia; 14 University of Birmingham, Birmingham, UK; 15 Guy’s and St Thomas’ NHS Foundation Trust, London, UK; 16 School of Nursing, The Hong Kong Polytechnic University, Hong Kong, Hong Kong; 17 Sir Peter MacCallum Department of Oncology, University of Melbourne, Victoria, Australia; 18 Epworth Healthcare, Victoria, Australia

**Keywords:** Peripheral neuropathy, Brentuximab vedotin, Lymphoma, Patient care, Neurotoxicity, Delphi method, Consensus recommendations

## Abstract

**Purpose:**

Brentuximab vedotin (BV) represents a therapeutic advancement in the treatment of CD30-positive lymphomas, yet its clinical utility is often limited by BV-induced peripheral neuropathy (PN). Currently, evidence-based, BV-specific guidelines for the assessment and management of PN are scarce, resulting in variability in clinical practice. This study aimed to establish international expert consensus on the optimal assessment, monitoring and management strategies for BV-induced PN in patients with lymphoma.

**Methods:**

A modified Delphi panel was conducted, engaging a total of 13 experts from eight different countries and representing haematology, neurology, dermatology, nursing and allied health disciplines. Panellists participated in three rounds of structured surveys, with statements developed based on a targeted literature review and guidance from an expert steering committee. Consensus was defined as ≥ 70% agreement/disagreement. Consensus was achieved on 60/87 (69%) statements across domains including risk factor assessment, symptom monitoring, pharmaceutical and non-pharmaceutical management.

**Results:**

Key recommendations include routine risk profiling before BV initiation, use of validated assessment tools at baseline and throughout therapy, multidisciplinary collaboration, and early intervention for worsening PN. Areas of uncertainty and variability in PN management remain, particularly regarding management of intermediate-grade PN and optimal non-pharmacological strategies.

**Conclusion:**

These international consensus recommendations provide practical guidance for improving clinical management of BV-induced PN and highlight the importance of early identification, personalised care and multidisciplinary collaboration. Further research is needed to address persistent gaps and inform future clinical guidelines.

**Supplementary Information:**

The online version contains supplementary material available at https://doi.org/10.1007/s00520-026-11314-5.

## Introduction

Brentuximab vedotin (BV) is an antibody–drug conjugate composed of a CD30‑directed monoclonal antibody linked to the microtubule-disrupting agent monomethyl auristatin E, enabling selective targeting of CD30-expressing malignant cells [1, 2]. It is indicated for the treatment of classical Hodgkin lymphoma (cHL), primary cutaneous and systemic anaplastic large cell lymphoma (sALCL), peripheral T-cell lymphoma (PTCL) and cutaneous T-cell lymphoma (CTCL) [3, 4]. BV has demonstrated significant clinical efficacy for patients with CD30-positive haematologic malignancies, which are often associated with high disease burden and poor outcomes, across frontline and relapsed/refractory settings, offering improved benefits over conventional therapies [2, 5–10].

However, the clinical benefits of BV are limited by treatment-related adverse events, most notably peripheral neuropathy (PN), occurring in up to two‑thirds of patients [11]. The risk and severity of PN is related to the cumulative dose and duration of BV, alone or in combination with other neurotoxic agents (e.g. vinblastine) [11]. Extended courses of single-agent BV, such as for CTCL or sALCL, are associated with a greater severity and duration of PN compared with shorter regimens used with combination chemotherapy [11].

BV-induced PN typically manifests as sensory symptoms, including tingling, numbness, and burning sensations in a distal, symmetrical distribution affecting the hands and feet. Less frequently, motor PN may present with cramps and distal muscle weakness in the upper and lower extremities, and as autonomic PN features, such as orthostatic hypotension or gastrointestinal dysautonomia [11]. While often reversible, complete resolution may take months to years [11, 12]. Some patients experience persistent symptoms that negatively impact quality of life (QoL) [1, 11, 13].

In pivotal clinical trials and real-world studies, PN may lead to BV dose modifications and discontinuation, though modifications have not demonstrated impact on the therapeutic efficacy of BV [6, 12, 14]. Nonetheless, increased hospitalisation rates, emergency department visits and outpatient visits represent a substantial healthcare cost of PN associated with BV [15].

The American Society of Clinical Oncology (ASCO) and European Society of Medical Oncology (ESMO) have published guidelines on the prevention and management of chemotherapy-induced PN (CIPN) [16, 17]. Additionally, North American experts have recently published practical recommendations focused specifically on the management of BV-induced PN in patients with lymphoma [1]. However, a gap remains for internationally developed, nuanced, practical guidelines for clinicians managing BV-induced PN specifically. Published literature has highlighted that there is no gold standard tool for assessing, grading and monitoring BV‑induced PN [11]. BV dosing can range from 1.2 mg/kg every two weeks (e.g. BV + AVD regimen), to 1.8 mg/kg every 3 weeks (e.g. BV with etoposide, cyclophosphamide, doxorubicin, dacarbazine and dexamethasone [BrECADD], BV + CHP), and treatment duration depends on lymphoma type and patient response [3]. Therefore, specific pharmacologic management options, including dose modifications, remain unclear. Further uncertainty surrounds individual risk factors for PN [11]. While multidisciplinary teams play a key role in effective management of PN, there are management inconsistencies across settings [11].

The aim of this modified Delphi study was to establish international expert consensus on optimal strategies for assessment, monitoring and treatment of BV-induced PN in patients with lymphoma, while identifying areas for future research, ultimately providing practical recommendations for all healthcare professionals (HCPs) involved in the management of BV-induced PN.

## Materials and methods

### Steering committee and Delphi panellists

Three experts were invited to form an international steering committee (SC), comprised of a neurologist, haematology oncologist, and dermatology oncologist from Spain, Australia, and Brazil. SC members were identified and invited by Costello Medical, independently of the study sponsor, based on their clinical and academic expertise in BV-induced PN and lymphoma management. Selection aimed to ensure representation across specialties and geography, considering factors including relevant publications, professional society involvement, guideline development activities and clinical experience in the field. The SC reviewed the Delphi panel protocol, approved the scope, and led the development of Delphi panel statements. The SC did not participate in consensus voting.

Eligible panellists were individuals with clinical or academic expertise in treating patients with PN; selection aimed to ensure multidisciplinary representation across professions involved in the recognition, assessment and management of BV-induced PN, including neurology, haematology-oncology, dermatology-oncology, nursing and rehabilitation. Panellists were identified through targeted literature searches and guided by the SC. Recruitment was conducted in stages to achieve a target panel size of up to 15 participants while maintaining representation across predefined specialties. A target panel size of up to 15 participants was selected pragmatically, consistent with Delphi methodology, which emphasises purposive selection of relevant experts rather than formal statistical sample-size calculation [18, 19]. The target size was intended to provide sufficient multidisciplinary representation while remaining feasible for iterative consensus development across multiple rounds. In total, 26 experts were invited to participate via email, of whom 15 agreed to participate.

The study sponsor did not participate in the identification of the SC or panellists, development of the consensus statements, or voting.

### Study design

The Delphi methodology was selected as a recognised and robust process for eliciting expert consensus. The study was conducted and reported in line with ACCORD and CREDES guidelines [20, 21]. This study was not registered prospectively.

Surveys were distributed using the Costello Medical Delphi App, a bespoke web-based application for consensus voting. Statements comprised of 6-point Likert scales (strongly agree, agree, slightly agree, slightly disagree, disagree or strongly disagree), multiple-choice, and open-response questions. A ‘not applicable’ response option was provided to allow panellists an option when a statement fell outside their expertise. Panellists were invited to contextualise their responses with free-text comments.

A pre-defined consensus threshold of ≥ 70% of respondents was set, in agreement with the SC [22]. For Likert-scale items, consensus was reached when ≥ 70% of respondents selected ‘strongly agree/agree’ or ‘strongly disagree/disagree’. ‘Slightly agree’ and ‘slightly disagree’ responses were considered neutral for consensus calculations. For multiple-choice questions, consensus was defined as ≥ 70% of panellists selecting a single option. Panellists who selected ‘not applicable’ in a specific statement were removed from the consensus calculations. Consensus was not calculated for open-response questions.

Statements were grouped into the following topics: the relationship between BV and PN, the diagnostic process and consideration for PN, pharmaceutical management of BV-induced PN, non-pharmaceutical management of BV-induced PN and future directions and research priorities.

Round 1 statements were developed using findings from a targeted literature review (TLR) and guidance from the SC. Targeted searches were conducted in PubMed to identify literature published from 2014–2024 that described the management of PN in patients with lymphoma treated with BV, with a focus on practical management. Published evidence from the TLR was used to identify key themes, areas of clinical uncertainty and evidence gaps relevant to the management of BV-induced PN, which informed the development of Round 1 statements. A summary of the key evidence informing statement development across each topic area is provided in Supplementary Table 1.

Statements in Rounds 2 and 3 were informed by the results, free-text responses and input from the SC based on the preceding round(s). Statements that achieved consensus were removed from subsequent rounds. Non-consensus statements were reviewed by the SC alongside panellists' free-text comments. Statements close to consensus were typically rephrased and re-evaluated. Statements with lower levels of agreement were generally not carried forward unchanged; however, where the topic was considered clinically important, substantially revised or new statements informed by panellist feedback could be added in subsequent rounds. Panellists could view the anonymised aggregated response from the preceding round for related statements.

No patients were involved in the study, which was not submitted for approval by an ethics committee or institutional review board. Panellists gave informed consent to participate in the study.

## Results

Of the 15 individuals who agreed to participate, 13 experts completed all three rounds: three haematologists, three neurologists, one clinical researcher specialising in PN, three nurses, two dermatology oncologists, and one occupational therapist (OT)/physiotherapist. This group represented experts from Asia and the Pacific (Hong Kong, Australia), Europe (Spain, Italy, United Kingdom, Greece, Norway), and South America (Brazil). Two invited individuals did not complete the Round 1 survey and were excluded from subsequent rounds.

**Fig. 1:**
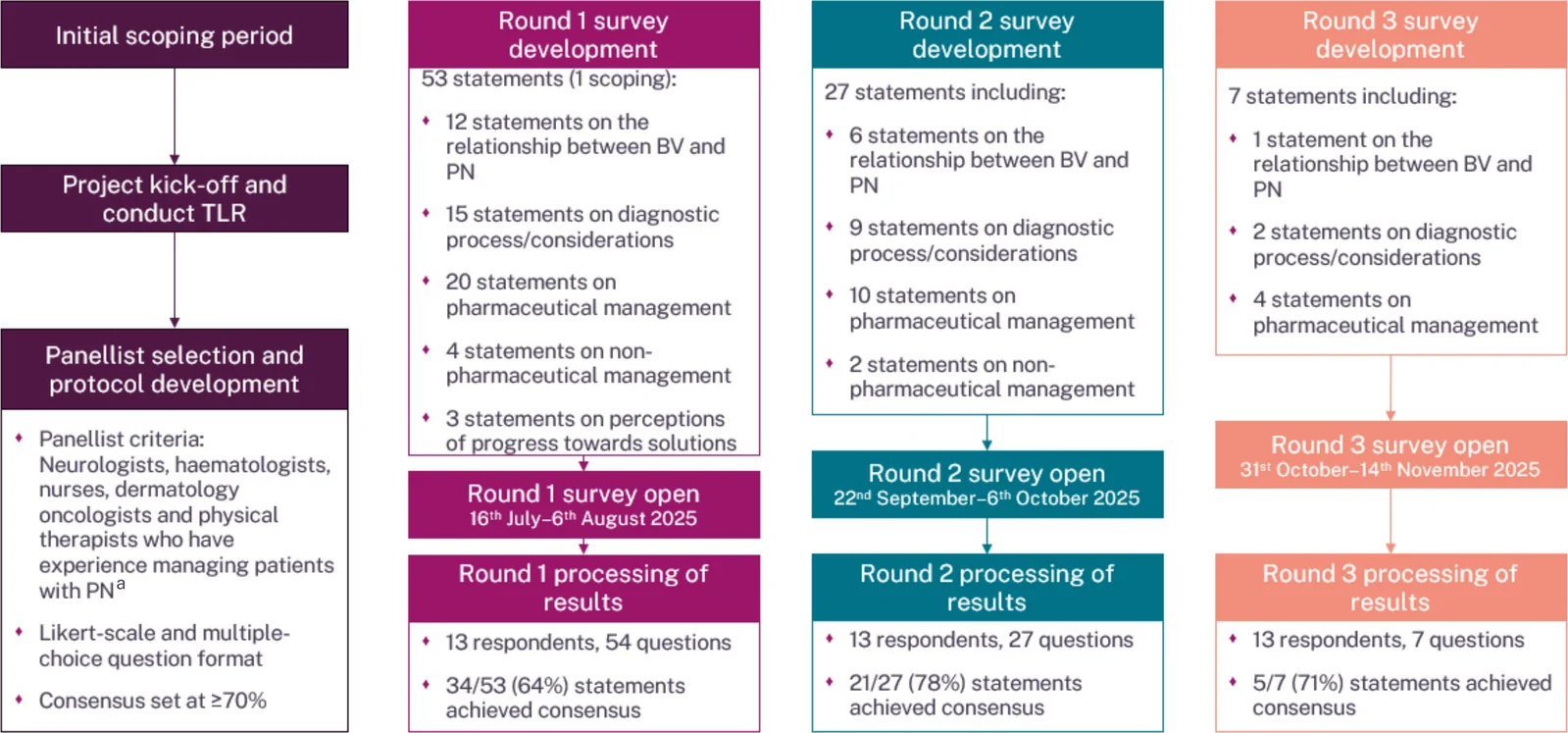
Overview of modified Delphi panel results. ^a^Experts were identified through publication history, clinical experience, professional society involvement and Steering Committee recommendation. For Likert-scale items, consensus was reached when ≥ 70% of respondents selected ‘strongly agree/agree’ or ‘strongly disagree/disagree’. BV: brentuximab vedotin; PN: peripheral neuropathy; TLR: targeted literature review

Three survey rounds were conducted between 16th July and 14th November 2025 (Fig. 1). Across all three rounds, 60/87 (69%) statements measuring agreement reached consensus (34/53 [64%] in Round 1; 21/27 [78%] in Round 2 and 5/7 [71%] in Round 3); one statement in Round 1 was a scoping question, with free-text responses. A full list of statements and their progression across rounds is presented in Supplementary Tables 2‒6. A full list of statements reaching consensus is presented in Table 1.

**Table 1:** All statements that reached consensus

Statement	Outcome; n/N (% of panellists)	Round
**Relationship between BV and PN**		
In your experience, please select any risk factors that you would associate with brentuximab vedotin induced peripheral neuropathy for patients with lymphoma	Age; 8/13 (62) Clinical stage of lymphoma; 3/13 (23) **Prior neurotoxic therapy (e.g. vincristine); 10/13 (77)** **Current neurotoxic therapy (e.g. vinblastine); 10/13 (77)** **High cumulative brentuximab vedotin dose; 11/13 (85)** **Comorbidities (e.g. diabetes); 11/13 (85)** Hereditary neuropathies; 9/13 (69) Other (please specify); 1/13 (8)	1
Risk factor profiling should be conducted routinely prior to initiation of brentuximab vedotin in patients with lymphoma	**Agree; 12/13 (92)** Disagree; 0/13 (0)	1
Risk factor profiling should include an assessment of patient and family history of hereditary neuropathies	**Agree; 11/13 (85)** Disagree; 1/13 (8)	1
Healthcare professionals should discuss the potential risks of peripheral neuropathy with patients prior to initiating brentuximab vedotin to allow them to make informed decisions on their treatment course	**Agree; 11/13 (85)** Disagree; 0/13 (0)	1
Part 1: In your experience, please select any early indicators of escalating peripheral neuropathy which would alert you to neuropathic effects of brentuximab vedotin.^*^	Sensitivity to touch; 8/13 (62) Sensitivity to temperature; 7/13 (54) **Progressive sensation loss; 10/13 (77)** **Paraesthesia (i.e. pins and needles); 11/13 (85)** Neuropathic pain; 9/13 (69) Falls due to loss of balance; 9/13 (69) Loss of reflexes; 6/13 (46)	1
Part 1: In your experience, please select any indicators of escalating peripheral neuropathy which should warrant immediate intervention (i.e. dose modification) to prevent irreversible peripheral nerve damage.^*^	Sensitivity to touch; 2/13 (15) Sensitivity to temperature; 2/13 (15) Progressive sensation loss; 9/13 (69) Paraesthesia (i.e. pins and needles); 7/13 (54) Neuropathic pain; 8/13 (62) **Falls due to loss of balance; 11/13 (85)** Loss of reflexes; 6/13 (46)	1
Part 2: In your experience, please select any indicators of escalating peripheral neuropathy which should warrant immediate intervention (i.e. dose modification) to prevent irreversible peripheral nerve damage.^*^	**Distal weakness; 10/13 (77)** Muscle cramping; 4/13 (31) Constipation/diarrhoea; 4/13 (31) Orthostatic hypertension; 8/13 (62) Other (please specify); 2/13 (15)	1
A patient who has pre-existing Grade 3 and above peripheral neuropathy should be excluded from receiving brentuximab vedotin	**Agree; 11/12 (92)** Disagree; 0/12 (0)	2
The decision to exclude a patient who has persistent/ongoing peripheral neuropathy from receiving brentuximab vedotin should be made on a case-by-case basis, including consideration of the severity of prior neuropathy, lifestyle and the availability of alternative treatments	**Agree; 13/13 (100)** Disagree; 0/13 (0)	2
Progressive sensation loss^†^ is an indicator of escalating peripheral neuropathy which should warrant immediate consideration of intervention (i.e. dose modification) to prevent irreversible peripheral nerve damage	**Agree; 10/12 (83)** Disagree; 0/12 (0)	2
The relationship between lymphoma type (cutaneous vs. non-cutaneous) and prevalence/severity of brentuximab vedotin-induced peripheral neuropathy is unclear. Future empirical studies are needed to explore this potential association	**Agree; 8/10 (80)** Disagree; 0/10 (0)	2
It is unlikely that brentuximab vedotin-induced peripheral neuropathy at Grade 2 and above will stabilise spontaneously, if brentuximab vedotin is continued without dose modification or dose delay	**Agree; 10/12 (83)** Disagree; 0/12 (0)	2
In addition to the most frequent clinical presentations of peripheral neuropathy, healthcare professionals should remain vigilant for common symptoms of autonomic nerve damage (e.g. orthostatic hypotension and constipation/diarrhoea) as potential early indicators of brentuximab vedotin-induced peripheral neuropathy	**Agree; 11/13 (85)** Disagree; 0/13 (0)	3
**Diagnostic processes and considerations for BV-induced PN**		
Early diagnosis of peripheral neuropathy is the most effective way to manage its severity	**Agree; 12/13 (92)** Disagree; 1/13 (8)	1
To encourage patients to report symptoms as early as possible, healthcare professionals should emphasise to the patient that early detection of peripheral neuropathy allows for prolonged treatment with brentuximab vedotin. This would help to address patient fears around stopping or reducing brentuximab vedotin treatment, affecting their overall treatment efficacy and outcomes	**Agree; 11/13 (85)** Disagree; 1/13 (8)	1
There is a need for resources to help educate patients on peripheral neuropathy symptoms to ensure timely reporting	**Agree; 12/13 (92)** Disagree; 1/13 (8)	1
There should be careful consideration of the patient’s job and lifestyle (e.g. hobbies or activities that involve dexterity) when discussing the implications of peripheral neuropathy on their quality of life	**Agree; 12/13 (92)** Disagree; 0/13 (0)	1
Healthcare professionals should remain alert to the risk of underreporting neuropathy symptoms, particularly in patients with poor prognoses of lymphoma	**Agree; 10/13 (77)** Disagree; 0/13 (0)	1
Healthcare professionals should ask patients about symptoms and signs of neuropathy at each patient visit	**Agree; 13/13 (100)** Disagree; 0/13 (0)	1
At first presentation of neuropathy symptoms, the patient should be evaluated for peripheral neuropathy using a validated diagnostic tool (e.g. the Common Terminology Criteria for Adverse Events [CTCAE] scale, the Total Neuropathy Score [TNS])	**Agree; 10/13 (77)** Disagree; 1/13 (8)	1
Peripheral neuropathy should be measured using a validated diagnostic tool at least every 3 cycles of brentuximab vedotin	**Agree; 10/13 (77)** Disagree; 1/13 (8)	1
The TNS is an effective method of diagnosing and grading brentuximab vedotin-induced peripheral neuropathy	**Agree; 11/12 (92)** Disagree; 0/12 (0)	1
In your experience, what are the indicators for organising nerve conduction testing?	Rapid progress of neuropathy; 9/13 (69) **Severe neuropathy; 11/13 (85)** **Atypical presentation; 10/13 (77)** **Motor neuropathy symptoms; 10/13 (77)** Other; 1/13 (8)	1
Typically, escalation from mild peripheral neuropathy to severe peripheral neuropathy happens gradually (over the course of ≥ 2 cycles of brentuximab vedotin)	**Agree; 10/12 (83)** Disagree; 0/12 (0)	1
During treatment, under which of the following circumstances should the patient be referred for a neurology consultation?	Very early onset neuropathy; 6/13 (46) **Rapid progression of neuropathy; 12/13 (92)** **Atypical course of neuropathy; 13/13 (100)** **Uncontrolled neuropathic pain; 11/13 (85)** **Motor weakness; 11/13 (85)** Grade 3‒4 neuropathy; 8/13 (62) Other (please specify); 1/13 (8)	1
Patients should be asked about their peripheral neuropathy symptoms using a patient-reported outcome measure (PROM; e.g. QLQ-CIPN20) at every visit	**Agree; 10/13 (77)** Disagree; 0/13 (0)	2
Prior to initiating brentuximab vedotin, patients with pre-existing peripheral neuropathy (regardless of the cause) should undergo a baseline neuropathy assessment by a neurologist using the TNSc, supplemented with patient-reported outcome measures	**Agree; 11/13 (85)** Disagree; 1/13 (8)	2
Peripheral neuropathy should be measured using the TNSc at baseline and at least once every three cycles of brentuximab vedotin	**Agree; 10/13 (77)** Disagree; 1/13 (8)	2
The TNSc is a useful tool for the monitoring and follow-up of peripheral neuropathy in patients treated with brentuximab vedotin, allowing clinicians to track changes over time	**Agree; 11/12 (92)** Disagree; 0/12 (0)	2
The TNSc is a useful complementary tool for diagnosing and grading brentuximab vedotin-induced peripheral neuropathy, that should be considered as an adjunct to the CTCAE and PROMs, especially in the context of clinical trial design	**Agree; 12/12 (100)** Disagree; 0/12 (0)	2
If available, a nerve conduction study should be considered in which of the following scenarios?	Prior to treatment, if the patient has a history of peripheral neuropathy that has not been recently re-evaluated; 5/13 (38) Prior to treatment, if the patient has an abnormal TNSc, in order to rule out subclinical peripheral neuropathy; 7/13 (54) During treatment, if the patient develops rapidly progressive sensory-motor or motor peripheral neuropathy, to rule out a Guillain-Barré syndrome-like presentation of brentuximab vedotin-induced neuropathy; 8/13 (62) **During or after treatment, in cases of diagnostic uncertainty regarding the origin of symptoms due to atypical presentation (e.g. limited to the upper extremities, asymmetry); 10/13 (77)** After treatment, if the patient continues to present with moderate-to-severe neuropathy and/or shows no improvement; 8/13 (62) None of the above; 1/13 (8)	2
Who should primarily oversee diagnosing and grading of peripheral neuropathy induced by brentuximab vedotin?	Nurse; 4/13 (31) **Treating physician; 11/13 (85)** Neurologist; 7/13 (54) Other [please specify]; 2/13 (15)	2
If available, a nerve conduction study should be considered during or after treatment, in cases of diagnostic uncertainty regarding the origin of symptoms due to atypical presentation (e.g. limited to the upper extremities, asymmetry) or in cases where there is suspicion of a Guillain-Barré syndrome-like presentation	**Agree; 13/13 (100)** Disagree; 0/13 (0)	3
**Pharmaceutical management of BV-induced PN**		
Dose reduction (i.e. reducing the amount of drug administered) is more effective at reducing peripheral neuropathy than prolonging the interval between doses	**Agree; 9/11 (82)** Disagree; 0/11 (0)	1
In the circumstance where a patient has baseline neuropathy, healthcare professionals may consider initiating brentuximab vedotin at a lower dose than usually recommended	**Agree; 10/13 (77)** Disagree; 1/13 (8)	1
For patients with Grade 1 peripheral neuropathy, no dose reductions or modifications of brentuximab vedotin are required	**Agree; 10/13 (77)** Disagree; 1/13 (8)	1
For patients with Grade 2 peripheral neuropathy on a regimen of brentuximab vedotin (1.8 mg/kg) with cyclophosphamide, doxorubicin and prednisone (CHP), as used for peripheral T-cell lymphoma (ECHELON-2 trial), brentuximab vedotin dose should be reduced to 1.2 mg/kg up to a maximum of 120 mg every three weeks	**Agree; 7/10 (70)** Disagree; 1/10 (10)	1
For patients with Grade 3 peripheral neuropathy on a regimen of brentuximab vedotin (1.2 mg/kg) with doxorubicin, vinblastine and dacarbazine (AVD), as used for Hodgkin lymphoma (ECHELON-1 trial), brentuximab vedotin dose should be held until improvement to Grade 2 or lower, then restarted at 0.9 mg/kg up to a maximum of 90 mg every 2 weeks	**Agree; 8/10 (80)** Disagree; 0/10 (0)	1
For patients with Grade 4 peripheral neuropathy, brentuximab vedotin treatment should be discontinued	**Agree; 12/13 (92)** Disagree; 0/13 (0)	1
Given their potential to exacerbate neuropathy, patients undergoing treatment with brentuximab vedotin should be carefully monitored to avoid excessive intake of supplements containing pyridoxine (vitamin B6)	**Agree; 8/11 (73)** Disagree; 2/11 (18)	1
In your experience, patient choice and lifestyle are primary considerations in deciding whether to prescribe pain management medications for brentuximab-induced peripheral neuropathy	**Agree; 12/13 (92)** Disagree; 1/13 (8)	1
Patient choice is an important aspect of pharmaceutical management of brentuximab vedotin-induced peripheral neuropathy. Clinicians should discuss the available management options with patients during appointments to facilitate shared decision-making	**Agree; 13/13 (100)** Disagree; 0/13 (0)	1
In the circumstance where a patient has several risk factors for peripheral neuropathy, healthcare professionals may still consider initiating brentuximab vedotin at the standard dose, with careful monitoring of the patient’s neurological status	**Agree; 12/13 (92)** Disagree; 1/13 (8)	2
When deciding whether to re-treat a patient with the standard recommended brentuximab vedotin dose after they have previously experienced brentuximab vedotin-induced peripheral neuropathy, clinicians should first consider the patient's disease prognostic factors (e.g. disease stage) and the availability of alternative treatments	**Agree; 11/13 (85)** Disagree; 0/13 (0)	2
For patients with Grade 3 motor peripheral neuropathy on a regimen of brentuximab vedotin (1.8 mg/kg) with cyclophosphamide, doxorubicin and prednisone (CHP), as used for peripheral T-cell lymphoma (ECHELON-2 trial), brentuximab vedotin dose should be discontinued or withheld until symptoms reduce to Grade 2 or below	**Agree; 7/10 (70)** Disagree; 0/10 (0)	2
For patients with Grade 2 and above peripheral neuropathy on a regimen of brentuximab vedotin (1.2 mg/kg) with doxorubicin, vinblastine and dacarbazine (AVD), as used for previously untreated Hodgkin lymphoma (ECHELON-1 trial), vinblastine dose modification may be considered before adjusting the dose of brentuximab vedotin	**Agree; 7/9 (78)** Disagree; 0/9 (0)	2
Clinical decisions regarding brentuximab vedotin dose modifications in response to peripheral neuropathy should be based on a combination of patient choice and clinician expertise	**Agree; 11/13 (85)** Disagree; 0/13 (0)	2
Pain medications such as duloxetine, pregabalin and gabapentin may be considered for pain management in brentuximab vedotin-induced peripheral neuropathy, though the effectiveness of these agents is variable	**Agree; 12/13 (92)** Disagree; 0/13 (0)	2
Immunosuppressive therapies (e.g. corticosteroids, intravenous immunoglobulin) may be considered for patients with brentuximab vedotin-induced Guillain–Barre Syndrome, confirmed by a nerve conduction study.^‡^	**Agree; 8/10 (80)** Disagree; 1/10 (10)	2
In patients with prior brentuximab vedotin-induced peripheral neuropathy, the decision to re-treat with the standard dose should be guided by both the severity of any current neuropathy and by the history of past neuropathy	**Agree; 13/13 (100)** Disagree; 0/13 (0)	3
In my clinical experience, there are circumstances in which the management of brentuximab vedotin dosage for patients with Grade 2 peripheral neuropathy receiving brentuximab vedotin 1.8 mg/kg as monotherapy may differ from the approach described in the Summary of Product Characteristics (SmPC)	**Agree; 10/12 (83)** Disagree; 0/12 (0)	3
Intravenous immunoglobulin or plasma exchange may be considered for patients diagnosed with brentuximab vedotin-induced Guillain–Barre Syndrome, confirmed by a nerve conduction study.^‡^	**Agree; 11/11 (100)** Disagree; 0/11 (0)	3
**Non-pharmaceutical management of BV-induced PN**		
Non-pharmaceutical interventions should be integrated into treatment plans for brentuximab vedotin-induced peripheral neuropathy as standard practice, to be guided by a multidisciplinary team including specialists beyond neurologists, haematologists, dermatologists and nurses	**Agree; 12/13 (92)** Disagree; 0/13 (0)	1
In your experience, please select the non-pharmaceutical interventions that you have found to be effective for alleviating symptoms of brentuximab vedotin-induced peripheral neuropathy	Acupuncture; 6/13 (46) Vitamin B supplementation; 2/13 (15) Cannabinoids; 3/13 (23) **Physical therapy; 10/13 (77)** Oncology massage; 5/13 (38) Cognitive behavioural therapy; 3/13 (23) Cryotherapy; 1/13 (8) Transcutaneous electrical nerve stimulation (TENS); 3/13 (23) Other [please specify]; 3/13 (23)	1
There is a need for more research on the efficacy of non-pharmaceutical interventions for brentuximab vedotin-induced peripheral neuropathy	**Agree; 12/13 (92)** Disagree; 0/13 (0)	1
Physical and occupational therapists can provide non-pharmaceutical interventions to improve the physical quality of life in patients with brentuximab vedotin-induced peripheral neuropathy	**Agree; 13/13 (100)** Disagree; 0/13 (0)	2
Psychologists and counsellors can provide non-pharmaceutical support to address the psychological impact of brentuximab vedotin-induced peripheral neuropathy on patients’ quality of life	**Agree; 10/13 (77)** Disagree; 2/13 (15)	2
**Future directions and research opportunities**		
In your experience, please select from the following list the aspects of brentuximab vedotin-induced peripheral neuropathy where there is a need for clear guidelines	**Diagnosis; 10/13 (77)** Dose modification; 9/13 (69) Dose delay; 8/13 (62) **Pharmaceutical management; 11/13 (85)** **Non-pharmaceutical management; 11/13 (85)** Other [please specify]; 3/13 (23)	1
Further research is needed regarding the predictive factors of severe, irreversible peripheral neuropathy	**Agree; 12/13 (92)** Disagree; 0/13 (0)	1
More focus is needed on patient centricity when considering decisions around the management of brentuximab vedotin-induced peripheral neuropathy. To address this, there is a need to leverage patient and public involvement and engagement (PPIE) to ensure that patient needs are being addressed	**Agree; 10/13 (77)** Disagree; 1/13 (8)	1

Consensus was pre-defined as ≥ 70% panellists agreeing/disagreeing with a given statement. Agreement and disagreement were defined as those selecting ‘strongly agree’ or ‘agree’, and ‘strongly disagree’ or ‘disagree’, respectively; ‘slightly agree’ and ‘slightly disagree’ responses were considered neutral and are therefore not presented here. For multiple choice questions, consensus was reached where ≥ 70% panellists selected an option. Where panellists answered ‘not applicable’, their response was removed from the consensus calculation. ^*^The online voting platform had a restriction of 10 options for multiple-choice questions; these statements were split into two, to account for this. ^†^Progressive sensation loss refers to loss of sensation that moves proximally up the limbs. ^‡^While these statements stemmed from a Round 1 statement relating to pharmaceutical management, they were grouped under ‘diagnostic processes and considerations’ for Rounds 2 and 3, as they partially related to NCSs

*AVD* doxorubicin, vinblastine and dacarbazine, *BV* brentuximab vedotin, *CHP* cyclophosphamide, doxorubicin and prednisone, *CTCAE* Common Terminology Criteria for Adverse Events, *NCS* nerve conduction study, *PN* peripheral neuropathy, *PPIE* patient and public involvement and engagement, *PROM* patient-reported outcome measure, *QLQ-CIPN20* Quality of Life Questionnaire – Chemotherapy-Induced Peripheral Neuropathy, *SmPC* Summary of Product Characteristics, *TENS* transcutaneous electrical nerve stimulation, *TNSc* Total Neuropathy Score – clinical

### Exploring the relationship between BV and PN

Panellists identified several risk factors associated with BV-induced PN, including prior neurotoxic therapy (e.g. vincristine) (77%), current neurotoxic therapy (e.g. vinblastine) (77%), high cumulative BV dose (85%) and comorbidities (e.g. diabetes) (85%). There was consensus that routine risk factor profiling should be performed before initiating BV therapy (92%), including assessment of personal and family history of hereditary neuropathies (85%). Potential risks of PN should be discussed with patients prior to initiation of BV (85%).

Early indicators of neuropathy, including progressive sensation loss (77%) and paraesthesia (85%), were highlighted, and signs warranting immediate intervention, such as distal weakness (77%), loss of balance resulting in falls (85%), and rapid progression of sensation loss (83%).

Whether there was a relationship between lymphoma type (cutaneous vs non-cutaneous) and the prevalence (40%) and severity (30%) of BV-induced PN was unclear based on voting; panellists agreed that this area requires further research (80%). Consensus was reached that moderate-to-severe PN (Grade ≥ 2) is unlikely to resolve in the absence of BV dose adjustments (83%).

It was agreed that patients who have pre-existing Grade ≥ 3 PN should be excluded from receiving BV (92%), but that the decision to exclude a patient who has persistent/ongoing PN from receiving BV should be made on a case-by-case basis, including consideration of the severity of prior neuropathy, lifestyle and availability of alternative treatments (100%).

### Diagnostic process and considerations for PN

#### Early diagnosis

Consensus was reached that early diagnosis of PN is the most effective way to minimise severity and optimise patient outcomes (92%). The panel agreed that communicating to patients that early detection of PN can help them continue BV treatment longer encourages timely symptom reporting (85%), and that providing clear patient resources on PN symptoms is important for education (92%).

All panellists agreed that HCPs should ask patients about symptoms of PN at every visit (100%), while remaining alert to the risk of underreporting, particularly in patients with poorer prognoses (77%).

#### Assessment tools

There was consensus that validated tools should be used to assess PN at baseline and at least every three cycles of BV (77%), and employed at the first presentation of symptoms (77%). The Total Neuropathy Score – clinical (TNSc) was recognised as an effective method for diagnosing and grading BV-induced PN (92%), whereas consensus was not reached on the effectiveness of the Common Terminology Criteria for Adverse Events (CTCAE; 58%) [23, 24]. The panel supported the use of the TNSc as a diagnostic (100%) and monitoring tool (92%), particularly alongside the CTCAE and patient-reported outcomes. However, only half reported routinely using the TNSc in clinical practice (50%; Supplementary Table 3).

#### Key diagnostic considerations and escalation

Panellists noted that nerve conduction studies (NCS) are not routine for diagnosis of BV-induced PN, primarily due to feasibility and utility concerns for typical PN presentations. However, it was acknowledged that NCS should be considered in specific scenarios, such as severe neuropathy (85%), diagnostic uncertainty (77%), atypical presentations (77%), or suspected Guillain-Barré Syndrome (GBS; 100%). Immunomodulatory treatments, including intravenous immunoglobulin or plasma exchange, were supported in confirmed cases of BV-induced inflammatory demyelinating polyradiculoneuropathies, specifically GBS (100%).

There was consensus that progression from mild to severe PN generally occurs gradually over at least two treatment cycles (83%). The panel recognised the importance of considering patient lifestyle and occupation when discussing PN diagnosis and its impact on QoL (92%).

Consensus was not reached on the primary responsibility for diagnosing and grading PN lying with nurses (69% disagree; Supplementary Table 3); in Round 2, 85% of panellists agreed that this responsibility should fall to the treating physician. There was broad agreement that patients should be referred for neurology consultation during treatment if they show rapid progression of PN (92%), atypical neuropathy course (100%), uncontrolled neuropathic pain (85%), or motor weakness (85%).

### Pharmaceutical management and dose modifications for BV-induced PN

The panel reached consensus that BV dose reduction is more effective at mitigating PN than prolonging the interval between doses (82%). For patients with baseline PN, starting BV at a lower dose was considered appropriate (77%); consensus was not reached for initiating at a lower dose based on multiple risk factors (69%).

#### Recommendations by PN grade and BV regimen

Consensus was reached that, for patients with Grade 1 PN, no dose modifications of BV are required (77%). For Grade 2 PN on a regimen of BV + CHP, dose reduction from 1.8 mg/kg to 1.2 mg/kg up to a maximum of 120 mg every three weeks was supported (70%). In patients with Grade ≥ 2 PN on BV + AVD, modification of vinblastine dose may be considered before adjusting the dose of BV (78%). The panel expressed that, in their experience, management of BV dosage for patients with Grade 2 PN on monotherapy may sometimes differ from the Summary of Product Characteristics (SmPC), highlighting the need for practical flexibility (83%).

For Grade 3 PN on BV + AVD (HL), withholding BV until improvement to Grade ≤ 2 before resuming at a reduced dose of 0.9 mg/kg (maximum 90 mg every two weeks) reached consensus (80%). There was no consensus for recommendations for Grade 3 sensory neuropathy on BV + CHP and as a monotherapy (Supplementary Table 4). Consensus was reached that, for Grade 4 PN, BV should be permanently discontinued (92%).

#### Retreatment after PN

Consensus was reached that decisions on whether BV retreatment should occur at the standard dose for patients with a history of PN should be guided by both the current severity and the patient’s past experience of PN (100%). It was emphasised that clinicians should consider the patient’s prognosis and the availability of alternative treatments (85%).

#### Patient involvement and pain management

Panel members agreed that patient choice is central to decision-making regarding BV dose modifications (100%), and that shared decision-making, incorporating both patient preferences and clinical expertise, is essential (85%). Patient lifestyle was regarded as an important factor in pain management decisions (92%). The panel agreed that analgesics, including duloxetine, pregabalin, and gabapentin, may be considered for managing BV-induced neuropathic pain, but recognised variability in their effectiveness (92%). There was a lack of consensus on whether the use of pain medications during BV treatment risks masking PN symptoms, limiting its monitoring, or on whether pain or symptomatic medications should be considered only once PN is established (Supplementary Table 4).

#### Other drug-related considerations

There was consensus that patients receiving BV should avoid excessive intake of pyridoxine (vitamin B6), given its potential to exacerbate PN (73%).

### Non-pharmaceutical management of BV-induced PN

Physical therapy was identified as an effective non-pharmaceutical option for alleviating symptoms of BV-induced PN (77%). All panellists agreed that physiotherapists and OTs can provide interventions that improve physical QoL for patients with BV-induced PN (100%). Additionally, psychologists and counsellors play a key role in supporting the psychological wellbeing of these patients (77% agree). Panellists noted in free-text responses that nurses, treating physicians and social workers involved in routine oncology care may support provision of accessible psychological support.

There was consensus on the need for further research to evaluate the efficacy of other non-pharmaceutical interventions for BV-induced PN (92%).

### Future directions and research priorities

All three statements on progress towards clarifying the management of BV-induced PN in Round 1 reached consensus. The panel highlighted the need for clear, evidence-based guidelines on the diagnosis (77%), pharmaceutical management (85%) and non-pharmaceutical management (85%) of BV-induced PN. There was consensus that additional research is needed to identify predictive factors for severe or irreversible PN (92%). It was agreed that a greater emphasis on patient-centred care is needed in management decisions concerning BV-induced PN, including the integration of patient and public involvement and engagement (77%).

## Discussion

### Early identification, patient education and routine assessment are critical for optimal management

Early identification and proactive management of risk factors are fundamental to minimising the burden of BV-induced PN. Current best practice and expert consensus recommend comprehensive risk factor profiling, including detailed patient and family histories, prior to initiating BV [16, 25].

Prompt recognition of escalating neuropathy is facilitated by the identification of early clinical “red flags,” which extend beyond the common sensory (e.g. paraesthesia, numbness) and motor symptoms (e.g. distal weakness, cramps) to include less frequently recognised autonomic manifestations such as orthostatic hypotension and constipation. Importantly, moderate-to-severe demyelinating BV-induced PN, although uncommon, requires specific multidisciplinary management approaches [26, 27].

Timely recognition of PN symptoms were emphasised as key elements of effective management, highlighting the need for regular, structured symptom assessment and ongoing patient education. Caregivers and families play a critical role in symptom recognition and care plan adherence, while patient reluctance to report PN symptoms, often due to concerns about losing access to BV and affecting their prognosis, can delay intervention [11, 28]. Equipping patients and caregivers with sufficient knowledge and resources (e.g. self-management information or symptom assessment tools) is essential to encourage early symptom reporting without compromising treatment efficacy.

**Fig. 2:**
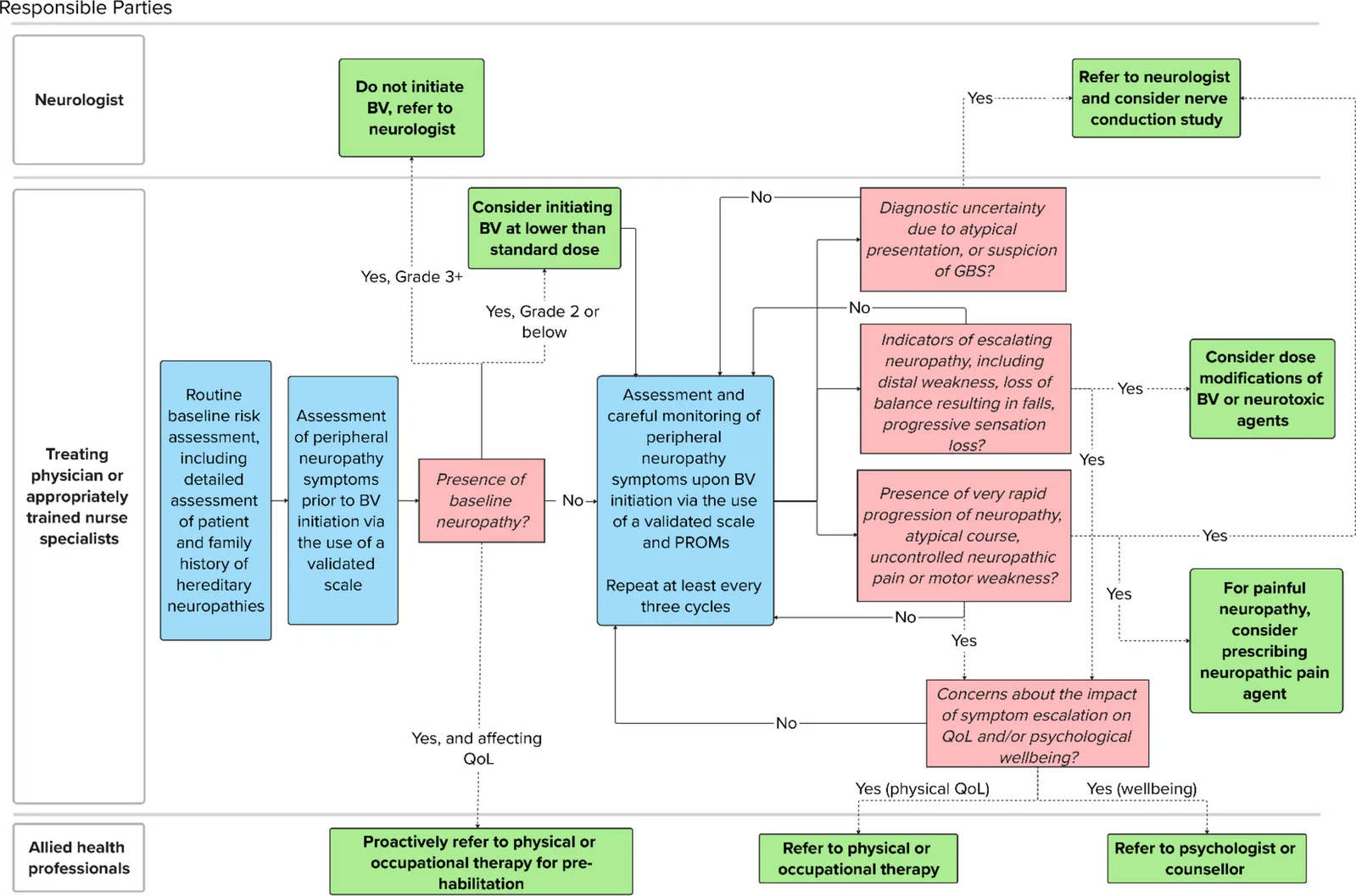
Management pathway for the assessment and monitoring of BV-induced PN. Dashed lines indicate circumstances where further action or escalation may be required. Abbreviations: BV: brentuximab vedotin; GBS: Guillain-Barré Syndrome; PN: peripheral neuropathy; QoL: quality of life

Oncology patients with high clinical complexity benefit significantly from care by specialist or advanced practice nurses, who provide individualised care and foster patient engagement in treatment decisions [29]; however, responsibilities across the management pathway may vary across settings. Key management practices, as established in this Delphi panel and previous literature, are shown in Fig. 2; these suggested practices serve as a blueprint which is adaptable to local settings.

### Achieving optimal management requires the use of robust, validated tools for assessment and monitoring

While the Delphi panel reaffirmed the value of employing robust, validated assessment tools to ensure timely detection and intervention, practical barriers may complicate implementation in real-world practice. The TNSc was recognised as a valuable instrument for assessing PN, considered complementary to patient-reported outcome measures such as the European Organisation for Research and Treatment of Cancer Quality of Life Questionnaire – CIPN (EORTC QLQ-CIPN20) [30]. Yet, routine implementation of the TNSc in clinical practice is often constrained by time and resource limitations.

The TNS-nurse, a streamlined tool (sensory, motor, autonomic, pin-prick and vibration), may be a practical alternative for symptom monitoring [31]. Nevertheless, panellists acknowledged that even short-form tools are difficult to integrate and implicate additional demands on limited nursing resources; previous expert consensus has indicated that questionnaires with more than three items may be too onerous in clinical practice [25].

Given these feasibility concerns, greater emphasis may be warranted on involving patients directly in the assessment of PN as part of broader symptom management. Patient-reported outcome tools, such as the QLQ-CIPN20 and Patient-Reported Outcomes CTCAE (PRO-CTCAE) scale, may be preferable alternatives or adjuncts to clinical assessment tools to improve treatment decision-making in routine care [32, 33].

### Agreement was reached on dose modification in some scenarios

While clear consensus was reached on the approaches to management of Grade 1 and 4 neuropathy, which aligned with product labelling, there was less consensus regarding intermediate CTCAE grades [3]. The management of Grade 3 PN in patients treated with BV, whether as monotherapy or in combination with CHP for sALCL and other CD30 + peripheral T-cell lymphomas, was a particular area of uncertainty.

Grade 3 and above PN is relatively uncommon, with an estimated prevalence of approximately 6% in patients with cHL [34]. Consequently, direct clinical experience in managing this level of severity is limited, and there is a lack of robust data on the rate and likelihood of symptom resolution compared to Grade 2 PN [11]. Patients at greatest risk of developing Grade 3 PN, such as those with high cumulative exposure to neurotoxic agents in the relapsed/refractory setting, typically have a poorer prognosis due to limited treatment options and advanced disease [6]. Free-text responses from the Delphi panel consistently recognised that Grade 3 PN warrants prompt intervention due to its significant impact on patients’ QoL, aligning with the SmPC for BV [3]. In this context, optimal management, whether dose reduction, delay, or discontinuation, should be tailored to prognosis, treatment stage and lymphoma subtype.

Similarly, varying approaches to managing Grade 2 neuropathy were highlighted, particularly when BV is used as a monotherapy, with panellists indicating that dose modification would be context-dependent. Free text responses indicated, for example, that it is more likely that the treating physician would modify dosing if Grade 2 PN occurred in the patient’s third cycle of treatment, compared with their penultimate one.

Contrary to the prescribing information label, when BV is used in combination with AVD, the panel agreed that vinblastine could be reduced before considering modifications to BV dose. Indeed, real-world evidence has suggested that, in practice, some HCPs will choose to modify vinblastine in this regimen (17% of patients) when managing PN [12]. Emerging trial evidence suggests that, for patients with early-stage cHL, omitting vinblastine doses (i.e. a regimen of BV + AD) resulted in a much-improved toxicity profile, with predominantly Grade 1 PN reported and no cases of Grade 3 and above neuropathy [35].

**Fig. 3:**
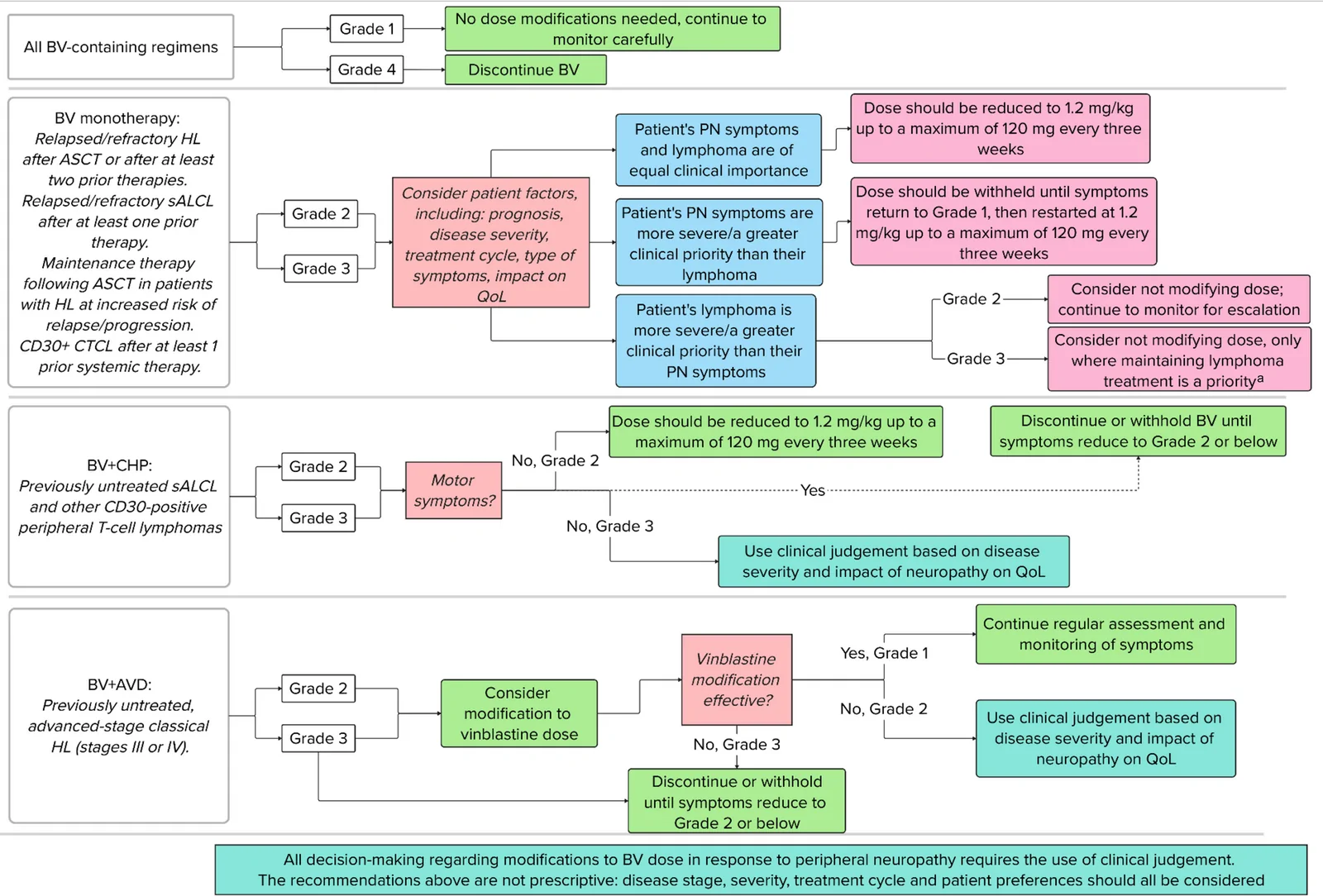
Dose modification management of BV-induced PN. ^a^For example, situations where the anticipated benefit of maintaining BV therapy for lymphoma control is judged to outweigh the risks associated with worsening PN (e.g. in patients with aggressive disease, limited treatment options, or where curative treatment intent is prioritised). Clinical assessment should include an assessment of patient risk, treatment indication (first-line versus last resort), disease type (cutaneous lymphoma or Hodgkin lymphoma) and treatment duration that the neuropathy was identified in (first versus penultimate cycle). BrECADD is recommended for previously untreated advanced stage classical HL; Delphi statements were not developed on this regimen, as this combination was approved after the finalisation of the study protocol. Thus, this combination has not been included in Fig. 3. Abbreviations: ASCT: autologous stem cell transplantation; AVD: doxorubicin, vinblastine, and dacarbazine; BrECADD: BV with etoposide, cyclophosphamide, doxorubicin, dacarbazine and dexamethasone; BV: brentuximab vedotin; CTCL: cutaneous T-cell lymphoma; HL: Hodgkin lymphoma; PN: peripheral neuropathy; QoL: quality of life; sALCL: systemic anaplastic large cell lymphoma

Recommendations for the management of Grade 2 and 3 PN, informed by findings from this Delphi study, prior literature, and clinical experience, are summarised in Fig. 3. In practice, dose modifications may differ from SmPC recommendations, where clinically appropriate. As the BrECADD combination regimen was approved after the finalisation of the Delphi protocol, suggested dose modifications for this regimen were not explored [3].

### Shared decision-making and patient-centred care are essential

The panel emphasised the importance of dialogue with patients about PN, including discussion of risk factors, early symptoms and the potential impact on daily functioning and QoL. The impact of PN is individual, and even lower grades of neuropathy may be significant for people reliant on fine motor skills or dexterity. For example, musicians, surgeons, artists or professionals reliant on precise hand movements may experience a disproportionate impact on their work and overall well-being, even at lower grades of neuropathy. When discussing the implications of PN, HCPs should be careful to consider this, including an emphasis on the risk of being unable to work, if applicable. HCPs should be mindful that it is often difficult for patients to envision the impact of PN on their life, if they have not experienced it before.

While it was noted that clinicians’ expertise should be used to inform decisions around dose modifications, a shared decision-making approach was emphasised by the panel. Shared decision-making ensures patients are fully informed about the risks and benefits of various management approaches, including the rationale behind dose modifications and interruptions.

### Supportive pharmacological and non-pharmaceutical approaches play a key role

Pain medications (e.g. duloxetine, pregabalin and gabapentin) may be considered for managing BV-induced PN; however, as robust evidence regarding their efficacy is limited, they should not be considered a primary solution for PN management [17].

The roles of physiotherapists, OTs and nurses in providing supportive care to enhance physical QoL was emphasised. Existing evidence demonstrates that exercise therapy is effective in managing CIPN, leading to improvements in physical function (including balance and strength) as well as reductions in pain and overall symptom burden [36]. OTs can support improvement in physical QoL symptoms with sensory re‑education and graded tactile tasks targeting somatosensory discrimination [37].

Given the emotional toll of PN, psychologists can help patients and families adapt and improve QoL [38]. Social workers may be appropriate in cases where severe PN leads to dependence or challenges in daily living [39].

Optimal referral timing to allied health professionals (AHPs) for psychological and/or physical QoL support was not explored in the panel. However, SC members emphasised a proactive referral, to ensure interventions are in place prior to the development of severe PN symptoms. Neurologists and the treating clinical team should be familiar with local referral pathways to AHPs, ensuring streamlined communication and coordinated patient care.

Overall, while non-pharmaceutical and multidisciplinary strategies are valued, more evidence is required to define their efficacy, best practices and specific points of intervention within the care pathway [16, 17].

### Limitations

While the Delphi method is valuable for areas lacking robust empirical evidence, recommendations are influenced by the subjective experiences and clinical practices of the panellists and should be interpreted within this context. Although the panel included a range of HCPs, pharmacists were not represented. Inclusion of pharmacists may have provided additional perspectives on pharmacological management and medication optimisation. Additionally, participants from the United States and Canada were excluded because separate activities focused on BV-induced PN management were understood to be ongoing in these regions at the time of study design. The study therefore sought to capture perspectives from other international regions and should not be interpreted as representing a global consensus. Although this Delphi panel includes representation across multiple countries outside of North America, recommendations may not be generalisable to all regions or healthcare systems. Finally, areas in which evidence is sparse, or consensus was not reached, highlight the need for further research to address remaining gaps.

## Conclusion

This Delphi panel addressed a key gap in the management of BV-induced PN management, establishing consensus on foundational principles for assessment and monitoring, management practices by dose modification and multidisciplinary support. Further research is required to clarify nuances in dose modification, efficacy of non-pharmaceutical interventions and referral pathways. However, these consensus recommendations lay the groundwork for future guideline development to optimise the management of BV-induced PN, with the ultimate goal of improving patient outcomes and QoL.

## Supplementary Information

Below is the link to the electronic supplementary material.

Supplementary file1 (DOCX 114 KB)

## Data Availability

Aggregated deidentified data supporting the findings of this study and study materials (surveys, protocol, and consensus definitions) are available from the corresponding author upon reasonable request. Individual responses are not available, due to confidentiality agreements with panellists.
