# Supplementary material for: Expert consensus on the management of peripheral neuropathy in patients with lymphoma on brentuximab vedotin
Source: Support Care Cancer. 2026 Oct 17;34(11):1116. doi: 10.1007/s00520-026-11314-5 (PMC13676650; doi:10.1007/s00520-026-11314-5)
Supplement: Supplementary file 1 [file 520_2026_11314_MOESM1_ESM.docx]

**Expert Consensus on the Management of Peripheral Neuropathy in Patients with Lymphoma on Brentuximab Vedotin**

Roser Velasco,^1,2^ José A Sanches,^3^ Andreas A Argyriou,^4^ Guido Cavaletti,^5,6^ Eva Domingo-Domenech,^7^ Alexander Fosså,^8^ Jaime Gállego Pérez-Larraya,^9^ Antonia Miranda Ruiz,^10^ Alex Molassiotis,^11^ André Neder Ramires Abdo,^3^ Pablo L Ortiz-Romero,^12^ Susanna B Park,^13^ Julia Scarisbrick,^14^ Lauren Urwin,^15^ Mian Wang,^16^ H Miles Prince^17,18^

^1^Department of Neurology, Institut Català d’Oncologia–Hospital Universitari de Bellvitge, Bellvitge Biomedical Research Institute (IDIBELL), L'Hospitalet, Barcelona, Spain; ^2^Department of Cell Biology, Physiology and Immunology, Centro de investigación Biomédica en Red Enfermedades Neurodegenrativas (CIBERNED), Universitat Autònoma de Barcelona, Bellaterra, Spain; ^3^University of Sao Paulo Medical School, Sao Paulo, Brazil; ^4^Agios Andreas General Hospital of Patras, Patras, Greece; ^5^Università di Milano-Bicocca, Monza, Italy; ^6^Fondazione IRCCS San Gerardo dei Tintori, Monza, Italy; ^7^Institut Catala d’Oncologia, Hospital Duran Reynals Barcelona, Bellvitge Biomedical Research Institute (IDIBELL), Barcelona, Spain; ^8^Oslo University Hospital, Oslo, Norway; ^9^Clínica Universidad de Navarra, Instituto de Investigación Sanitaria de Navarra (IDISNA), Pamplona, Spain; ^10^University Costa del Sol Hospital Marbella, Málaga, Spain; ^11^School of Psychology, Health & Clinical Sciences at Aston University, Birmingham, United Kingdom; ^12^University Hospital 12 de Octubre, Institute i+12 Medical School, University Complutense, Madrid, Spain; ^13^Faculty of Medicine and Health, The University of Sydney, Sydney, Australia; ^14^University of Birmingham, Birmingham, UK; ^15^Guy’s and St Thomas’ NHS Foundation Trust, London, United Kingdom; ^16^School of Nursing, The Hong Kong Polytechnic University, Hong Kong; ^17^Sir Peter MacCallum Department of Oncology, University of Melbourne, Victoria, Australia; ^18^Epworth Healthcare, Victoria, Australia

Supplementary Table 1. Key evidence themes informing Round 1 Delphi statement development

| **Topic area** | **Key themes identified from the targeted literature review** | **Representative references informing statement development** |
| --- | --- | --- |
| Relationship between BV and PN | - Incidence and clinical presentation of BV-induced PN - Reversibility of symptoms - Potential risk factors including cumulative dose, prior neurotoxic exposure and comorbidities - Impact on quality of life | - Abramson JS et al. Crit Rev Oncol Hematol. 2024;204:104499. - Corbin ZA et al. J Neurooncol. 2017;132:439-46. - Fu et al. iScience. 2023. - Oak E, Bartlett NL. Expert Opin Drug Saf. 2016;15:875-82. - Stein et al. J Clin Med. 2024;13:823 - Velasco R, Domingo-Domenech E, Sureda A. Cancers. 2021;13:6125. |
| Risk assessment and patient counselling | - Importance of baseline assessment - Risk factor profiling - Patient education and early symptom reporting | - Abramson JS et al. Crit Rev Oncol Hematol. 2024;204:104499. - Fanale et al. Targeted Oncology. 2015. - Nademanee A et al. Biol Blood Marrow Transplant. 2018;24:2354-2359. - Scarisbrick JJ. Future Oncol. 2017;13:2405-2411. - Velasco R, Domingo-Domenech E, Sureda A. Cancers. 2021;13:6125. |
| Assessment and monitoring of PN | - Use of CTCAE, TNSc and patient-reported outcome measures - Challenges in grading and monitoring PN - Need for routine assessment during treatment | - Abramson JS et al. Crit Rev Oncol Hematol. 2024;204:104499. - Alberti P et al. Neurology. 2021;97. - Cavaletti G et al. J Peripher Nerv Syst. 2007;12. - Clifford K et al. Clin J Oncol Nurs. 2018;22(4). - Corbin ZA et al. J J Neurooncol. 2017;132. - Mariotto S et al. Leuk Lymphoma. 2019. - Velasco R, Domingo-Domenech E, Sureda A. Cancers. 2021;13:6125. |
| Neurology referral and diagnostic investigations | - Differential diagnosis of PN - Role of nerve conduction studies - Identification of atypical neuropathy presentations and inflammatory neuropathies | - Matthys A et al. Eur J Neurol. 2024;e16285. - Mariotto S et al. Leuk Lymphoma. 2019. - Abramson JS et al. Crit Rev Oncol Hematol. 2024;204:104499. |
| Pharmaceutical management | - Dose reduction, treatment interruption and discontinuation strategies - Management across different BV-containing regimens | - Oak E, Bartlett NL. Expert Opin Drug Saf. 2016;15:875-82. - Clifford K et al. Clin J Oncol Nurs. 2018;22(4). - Velasco R, Domingo-Domenech E, Sureda A. Cancers. 2021;13:6125. - Bowers JT et al. Blood Adv. 2023;7:6630-8. - Connors JM et al. N Engl J Med. 2018;378:331-344. - Forero-Torres A et al. Blood. 2015;126:2798-804. - Duvic M et al. J Clin Oncol. 2015;33:3759-65. - Abramson JS et al. Crit Rev Oncol Hematol. 2024;204:104499. - Heneghan MB et al. JCO Oncol Pract. 2024. - FDA. ADCETRIS Prescribing Information. 2014.^a^ - EMA. Adcetris (brentuximab vedotin) Summary of Product Characteristics (SmPC). 2014.^a^ |
| Pain management | - Duloxetine, gabapentin, pregabalin, tricyclic antidepressants and topical treatments for neuropathic symptoms - Limited evidence base for symptom control in BV-induced PN specifically | - Abramson JS et al. Crit Rev Oncol Hematol. 2024;204:104499. - Bowers JT et al. Blood Adv. 2023;7:6630-8. - Clifford K et al. Clin J Oncol Nurs. 2018;22(4). - Loprinzi CL et al. J Clin Oncol. 2020;38. - Mariotto S et al. Leuk Lymphoma. 2019. - Stein et al. J Clin Med. 2024;13:823. - Velasco R, Domingo-Domenech E, Sureda A. Cancers. 2021;13:6125. |
| Non-pharmaceutical management | - Physical therapy, occupational therapy, exercise interventions and multidisciplinary care approaches for PN | - Abramson JS et al. Crit Rev Oncol Hematol. 2024;204:104499. - Bowers JT et al. Blood Adv. 2023;7:6630-8. - Clifford K et al. Clin J Oncol Nurs. 2018;22(4). - Mariotto S et al. Leuk Lymphoma. 2019. - Velasco R, Domingo-Domenech E, Sureda A. Cancers. 2021;13:6125. |
| Evidence gaps and future research priorities | - Lack of BV-specific guidance - Uncertainty regarding predictors of severe PN - Need for further evidence on supportive interventions and optimal monitoring tools | - Clifford K et al. Clin J Oncol Nurs. 2018;22(4). - Velasco R, Domingo-Domenech E, Sureda A. Cancers. 2021;13:6125. |

^a^The targeted literature review used to inform Round 1 statements was conducted before the 2025 and 2026 updates to the FDA and EMA label for BV, respectively. Round 1 statements were informed by findings from the targeted literature review and grey literature review and Steering Committee expertise. References listed are representative sources informing each topic area and are not intended to represent a direct one-to-one mapping between individual Delphi statements and supporting evidence. Subsequent-round statements were additionally informed by panellist feedback and Steering Committee review. **Abbreviations**: BV: brentuximab vedotin; CTCAE: Common Terminology Criteria for Adverse Events; EMA: European Medicines Agency; FDA: Food and Drug Administration; PN: peripheral neuropathy; SmPC: Summary of Product Characteristics; TNSc: Total Neuropathy Score – clinical.

Supplementary Table 2. Flowchart of statements on the relationship between BV and PN

| Round 1 | Outcome (% of panellists) |  | Round 2 | Outcome (% of panellists) |  | Round 3 | Outcome (% of panellists) |
| --- | --- | --- | --- | --- | --- | --- | --- |
| In your experience, please select any risk factors that you would associate with brentuximab vedotin induced peripheral neuropathy for patients with lymphoma. | Age (62)  Clinical stage of lymphoma (23)  **Prior neurotoxic therapy (e.g. vincristine) (77)**  **Current neurotoxic therapy (e.g. vinblastine) (77)**  **High cumulative brentuximab vedotin dose (85)**  **Comorbidities (e.g. diabetes) (85)**  Hereditary neuropathies (69)  Other (please specify) (8) |  | | | | | |
| In your experience, what grade of pre-existing neuropathy should exclude a patient from receiving brentuximab vedotin? (Grading according to the Common Terminology Criteria for Adverse Events [CTCAE] scale) | Grade 1 and above (0)  Grade 2 and above (46)  Grade 3 and above (46)^*^  Grade 4 (8)^*^  Other (please specify) (8) |  | A patient who has pre-existing Grade 3 and above peripheral neuropathy should be excluded from receiving brentuximab vedotin. | **Agree (92)**  Disagree (0) |  | | |
|  |  |  | The decision to exclude a patient who has persistent/ongoing peripheral neuropathy from receiving brentuximab vedotin should be made on a case-by-case basis, including consideration of the severity of prior neuropathy, lifestyle and the availability of alternative treatments. | **Agree (100)**  Disagree (0) |  | | |
| Part 1: In your experience, please select any early indicators of escalating peripheral neuropathy which would alert you to neuropathic effects of brentuximab vedotin.^†^ | Sensitivity to touch (62)  Sensitivity to temperature (54)  **Progressive sensation loss (77)^‡^**  **Paraesthesia (i.e. pins and needles) (85)**  Neuropathic pain (69)  Falls due to loss of balance (69)  Loss of reflexes (46) |  | | | | | |
| Part 2: In your experience, please select any early indicators of escalating peripheral neuropathy which would alert you to neuropathic effects of brentuximab vedotin.^†^ | Distal weakness (69)  Muscle cramping (54)  Constipation/diarrhoea (31)  Orthostatic hypertension (31)  Other (please specify) (15) |  | Healthcare professionals should be aware of autonomic symptoms (e.g. orthostatic hypotension and constipation/diarrhoea) as potential early indicators of brentuximab vedotin-induced peripheral neuropathy, provided that other common causes of these symptoms have been excluded. | Agree (58)  Disagree (17) |  | In addition to the most frequent clinical presentations of peripheral neuropathy, healthcare professionals should remain vigilant for common symptoms of autonomic nerve damage (e.g. orthostatic hypotension and constipation/diarrhoea) as potential early indicators of brentuximab vedotin-induced peripheral neuropathy. | **Agree (85)**  Disagree (0) |
| Part 1: In your experience, please select any indicators of escalating peripheral neuropathy which should warrant immediate intervention (i.e. dose modification) to prevent irreversible peripheral nerve damage.^†^ | Sensitivity to touch (15)  Sensitivity to temperature (15)  Progressive sensation loss (69)**^‡^**  Paraesthesia (i.e. pins and needles) (54)  Neuropathic pain (62)  **Falls due to loss of balance (85)**  Loss of reflexes (46) |  | Progressive sensation loss**^‡^** is an indicator of escalating peripheral neuropathy which should warrant immediate consideration of intervention (i.e. dose modification) to prevent irreversible peripheral nerve damage. | **Agree (83)**  Disagree (0) |  | | |
| Part 2: In your experience, please select any indicators of escalating peripheral neuropathy which should warrant immediate intervention (i.e. dose modification) to prevent irreversible peripheral nerve damage.^†^ | **Distal weakness (77)**  Muscle cramping (31)  Constipation/diarrhoea (31)  Orthostatic hypertension (62)  Other (please specify) (15%) |  | | | | | |
| Risk factor profiling should be conducted routinely prior to initiation of brentuximab vedotin in patients with lymphoma. | **Agree (92)**  Disagree (0) |  | | | | | |
| Risk factor profiling should include an assessment of patient and family history of hereditary neuropathies. | **Agree (85)**  Disagree (8) |  | | | | | |
| Healthcare professionals should discuss the potential risks of peripheral neuropathy with patients prior to initiating brentuximab vedotin to allow them to make informed decisions on their treatment course. | **Agree (85)**  Disagree (0) |  | | | | | |
| In your experience, prevalence of brentuximab vedotin-induced peripheral neuropathy symptoms differs for cutaneous versus non-cutaneous lymphomas. | Agree (40)  Disagree (30) |  | The relationship between lymphoma type (cutaneous vs. non-cutaneous) and prevalence/severity of brentuximab vedotin-induced peripheral neuropathy is unclear. Future empirical studies are needed to explore this potential association. | **Agree (80)**  Disagree (0) |  | | |
| In your experience, severity of brentuximab vedotin-induced peripheral neuropathy symptoms differs for cutaneous versus non-cutaneous lymphomas. Please provide further details to your response in the free-text box. | Agree (30)  Disagree (10) |  |  |  |  |  |  |
| In some cases, peripheral neuropathy stabilises in patients treated with brentuximab vedotin without intervention. In your experience, are there any common indicators for this? | N/A |  | It is unlikely that brentuximab vedotin-induced peripheral neuropathy at Grade 2 and above will stabilise spontaneously, if brentuximab vedotin is continued without dose modification or dose delay. | **Agree (83)**  Disagree (0) |  | | |

Consensus was pre-defined as ≥70% panellists agreeing/disagreeing with a given statement. For multiple choice questions, consensus was reached where ≥70% panellists selected an option. Where panellists answered “not applicable”, their response was removed from the consensus calculation. **Abbreviations:** BV: brentuximab vedotin; CTCAE: Common Terminology Criteria for Adverse Events; PN: peripheral neuropathy; N/A: not applicable. ^*^One participant selected both Grade 3 and Grade 4 for this question. ^†^The online voting platform had a restriction of 10 options for multiple-choice questions; these statements were split into two, to account for this. **^‡^**Progressive sensation loss refers to loss of sensation that moves proximally up the limbs.

Supplementary Table 3. Flowchart of statements on diagnostic processes and considerations for BV-induced PN.

| Round 1 | Outcome (% of panellists) |  | Round 2 | Outcome (% of panellists) |  | Round 3 | | Outcome (% of panellists) | |
| --- | --- | --- | --- | --- | --- | --- | --- | --- | --- |
| Early diagnosis of peripheral neuropathy is the most effective way to manage its severity. | **Agree (92)**  Disagree (8) |  | | | | | | | |
| To encourage patients to report symptoms as early as possible, healthcare professionals should emphasise to the patient that early detection of peripheral neuropathy allows for prolonged treatment with brentuximab vedotin. This would help to address patient fears around stopping or reducing brentuximab vedotin treatment, affecting their overall treatment efficacy and outcomes. | **Agree (85)**  Disagree (8) |  | | | | | | | |
| There is a need for resources to help educate patients on peripheral neuropathy symptoms to ensure timely reporting. | **Agree (92)**  Disagree (8) |  | | | | | | | |
| There should be careful consideration of the patient’s job and lifestyle (e.g. hobbies or activities that involve dexterity) when discussing the implications of peripheral neuropathy on their quality of life. | **Agree (92)**  Disagree (0) |  | | | | | | | |
| Healthcare professionals should remain alert to the risk of underreporting neuropathy symptoms, particularly in patients with poor prognoses of lymphoma. | **Agree (77)**  Disagree (0) |  | | | | | | | |
| Healthcare professionals should ask patients about symptoms and signs of neuropathy at each patient visit. | **Agree (100)**  Disagree (0) |  | Patients should be asked about their peripheral neuropathy symptoms using a patient-reported outcome measure (e.g. QLQ-CIPN20) at every visit. | **Agree (77)**  Disagree (0) |  | | | | |
| At first presentation of neuropathy symptoms, the patient should be evaluated for peripheral neuropathy using a validated diagnostic tool (e.g. the Common Terminology Criteria for Adverse Events [CTCAE] scale, the Total Neuropathy Score [TNS]). | **Agree (77)**  Disagree (8) |  | Prior to initiating brentuximab vedotin, patients with pre-existing peripheral neuropathy (regardless of the cause) should undergo a baseline neuropathy assessment by a neurologist using the TNSc, supplemented with patient-reported outcome measures. | **Agree (85)**  Disagree (8) |  | | | | |
| Peripheral neuropathy should be measured using a validated diagnostic tool at least every 3 cycles of brentuximab vedotin. | **Agree (77)**  Disagree (8) |  | Peripheral neuropathy should be measured using the TNSc at baseline and at least once every three cycles of brentuximab vedotin. | **Agree (77)**  Disagree (8) |  | | | | |
| The CTCAE scale is an effective method of diagnosing and grading brentuximab vedotin-induced peripheral neuropathy. | **Agree (58)**  Disagree (42) | ***Deprioritised*** | | | | | | | |
| The TNS is an effective method of diagnosing and grading brentuximab vedotin-induced peripheral neuropathy. | **Agree (92)**  Disagree (0) |  | I, or my colleagues, often use the TNSc in clinical practice to diagnose, grade and monitor brentuximab vedotin-induced peripheral neuropathy. | Agree (50)  Disagree (33) | ***Deprioritised*** | | | | |
|  |  |  | The TNSc is a useful complementary tool for diagnosing and grading brentuximab vedotin-induced peripheral neuropathy, that should be considered as an adjunct to the CTCAE and patient reported outcome measures (PROMs), especially in the context of clinical trial design. | **Agree (100)**  Disagree (0) |  | | | | |
|  |  |  | The TNSc is a useful tool for the monitoring and follow-up of peripheral neuropathy in patients treated with brentuximab vedotin, allowing clinicians to track changes over time. | **Agree (92)**  Disagree (0) |  | | | | |
| A nerve conduction assessment is only necessary once a patient is experiencing symptoms of brentuximab vedotin-induced peripheral neuropathy. | Agree (38)  Disagree (46) |  | If available, a nerve conduction study should be considered in which of the following scenarios? | Prior to treatment, if the patient has a history of peripheral neuropathy that has not been recently re-evaluated (38)  Prior to treatment, if the patient has an abnormal Total Neuropathy Score Clinical (TNSc), in order to rule out subclinical peripheral neuropathy (54)  During treatment, if the patient develops rapidly progressive sensory-motor or motor peripheral neuropathy, to rule out a Guillain-Barré syndrome-like presentation of brentuximab vedotin-induced neuropathy (62)  **During or after treatment, in cases of diagnostic uncertainty regarding the origin of symptoms due to atypical presentation (e.g. limited to the upper extremities, asymmetry) (77)**  After treatment, if the patient continues to present with moderate-to-severe neuropathy and/or shows no improvement (62)  None of the above (8) |  | | If available, a nerve conduction study should be considered during or after treatment, in cases of diagnostic uncertainty regarding the origin of symptoms due to atypical presentation (e.g. limited to the upper extremities, asymmetry) or in cases where there is suspicion of a Guillain-Barré syndrome-like presentation. | | **Agree (100)**  Disagree (0) |
| In your experience, what are the indicators for organising nerve conduction testing? | Rapid progress of neuropathy (69)  **Severe neuropathy (85)**  **Atypical presentation (77)**  **Motor neuropathy symptoms (77)**  Other (8) |  |  |  |  |  |  |  |  |
| The responsibility of diagnosing and grading peripheral neuropathy primarily lies with nurses. | Agree (8)  Disagree (69) |  | Who should primarily oversee diagnosing and grading of peripheral neuropathy induced by brentuximab vedotin? | Nurse (31)  **Treating physician (85)**  Neurologist (54)  Other [please specify] (15) |  | | | | |
| Typically, escalation from mild peripheral neuropathy to severe peripheral neuropathy happens gradually (over the course of ≥2 cycles of brentuximab vedotin). | **Agree (83)**  Disagree (0) |  | | | | | | | |
| During treatment, under which of the following circumstances should the patient be referred for a neurology consultation? | Very early onset neuropathy (46)  **Rapid progression of neuropathy (92)**  **Atypical course of neuropathy (100)**  **Uncontrolled neuropathic pain (85)**  **Motor weakness (85)**  Grade 3‒4 neuropathy (62)  Other [please specify] (8) |  | | | | | | | |

Consensus was pre-defined as ≥70% panellists agreeing/disagreeing with a given statement. For multiple choice questions, consensus was reached where ≥70% panellists selected an option. Where panellists answered “not applicable”, their response was removed from the consensus calculation. **Abbreviations**: BV: brentuximab vedotin; CTCAE: Common Terminology Criteria for Adverse Events; PN: peripheral neuropathy; PROM: patient-reported outcome measure; QLQ-CIPN20: Quality of Life Questionnaire – Chemotherapy-Induced Peripheral Neuropathy; TNSc: Total Neuropathy Score – clinical.

Supplementary Table 4. Flowchart of statements on the pharmaceutical management of BV-induced PN.

| Round 1 | Outcome (% of panellists) |  | Round 2 | Outcome (% of panellists) |  | Round 3 | Outcome (% of panellists) |
| --- | --- | --- | --- | --- | --- | --- | --- |
| Dose reduction (i.e. reducing the amount of drug administered) is more effective at reducing peripheral neuropathy than prolonging the interval between doses. | **Agree (82)**  Disagree (0) |  | | | | | |
| In the circumstance where a patient has several risk factors for neuropathy, healthcare professionals may consider initiating brentuximab vedotin at a lower dose than usually recommended. | Agree (69)  Disagree (8) |  | In the circumstance where a patient has several risk factors for peripheral neuropathy, healthcare professionals may still consider initiating brentuximab vedotin at the standard dose, with careful monitoring of the patient’s neurological status. | **Agree (92)**  Disagree (8) |  | | |
| In the circumstance where a patient has baseline neuropathy, healthcare professionals may consider initiating brentuximab vedotin at a lower dose than usually recommended. | **Agree (77)**  Disagree (8) |  | | | | | |
| In the circumstance where a patient has been previously treated with brentuximab vedotin and developed peripheral neuropathy, healthcare professionals may still consider retreatment with brentuximab vedotin at the normally recommended dose. | Agree (50)  Disagree (33) |  | In patients with prior brentuximab vedotin-induced peripheral neuropathy, re-treatment at the standard dose should be guided more by the severity of the current neuropathy than by the history of past neuropathy. | Agree (69)  Disagree (23) |  | In patients with prior brentuximab vedotin-induced peripheral neuropathy, the decision to re-treat with the standard dose should be guided by both the severity of any current neuropathy and by the history of past neuropathy. | **Agree (100)**  Disagree (0) |
|  |  |  | When deciding whether to re-treat a patient with the standard recommended brentuximab vedotin dose after they have previously experienced brentuximab vedotin-induced peripheral neuropathy, clinicians should first consider the patient's disease prognostic factors (e.g. disease stage) and the availability of alternative treatments. | **Agree (85)**  Disagree (0) |  | | |
| For patients with Grade 1 peripheral neuropathy, no dose reductions or modifications of brentuximab vedotin are required. | **Agree (77)**  Disagree (8) |  | | | | | |
| For patients with Grade 2 or 3 peripheral neuropathy using brentuximab vedotin (1.8 mg/kg) as a monotherapy, doses should be withheld until symptoms return to Grade 1, then restarted at 1.2 mg/kg up to a maximum of 120 mg every three weeks. | Agree (64)  Disagree (9) |  | For patients with Grade 2 peripheral neuropathy using brentuximab vedotin (1.8 mg/kg) as a monotherapy, doses should be withheld until symptoms return to Grade 1, then restarted at 1.2 mg/kg up to a maximum of 120 mg every three weeks. | Agree (50)  Disagree (17) |  | In my clinical experience, there are circumstances in which the management of brentuximab vedotin dosage for patients with Grade 2 peripheral neuropathy receiving brentuximab vedotin 1.8 mg/kg as monotherapy may differ from the approach described in the Summary of Product Characteristics (SmPC). | **Agree (83)**  Disagree (0) |
|  |  |  | For patients with Grade 3 peripheral neuropathy using brentuximab vedotin (1.8 mg/kg) as a monotherapy, doses should be withheld until symptoms return to Grade 2 or below, then restarted at 1.2 mg/kg up to a maximum of 120 mg every three weeks. | Agree (55)  Disagree (9) |  | In my clinical experience, there are circumstances in which the management of brentuximab vedotin dosage for patients with Grade 3 peripheral neuropathy receiving brentuximab vedotin 1.8 mg/kg as monotherapy may differ from the approach described in the SmPC. | Agree (50)  Disagree (17) |
| For patients with Grade 2 peripheral neuropathy on a regimen of brentuximab vedotin (1.8 mg/kg) with cyclophosphamide, doxorubicin and prednisone (CHP), as used for peripheral T-cell lymphoma (ECHELON-2 trial), brentuximab vedotin dose should be reduced to 1.2 mg/kg up to a maximum of 120 mg every three weeks. | **Agree (70)**  Disagree (10) |  | | | | | |
| For patients with Grade 3 sensory peripheral neuropathy on a regimen of brentuximab vedotin (1.8 mg/kg) with cyclophosphamide, doxorubicin and prednisone (CHP), as used for peripheral T-cell lymphoma (ECHELON-2 trial), brentuximab vedotin dose should be reduced to 1.2 mg/kg up to a maximum of 120 mg every three weeks. | Agree (40)  Disagree (40) |  | For patients with Grade 3 sensory peripheral neuropathy on a regimen of brentuximab vedotin (1.8 mg/kg) with cyclophosphamide, doxorubicin and prednisone (CHP), as used for peripheral T-cell lymphoma (ECHELON-2 trial), brentuximab vedotin dose should be withheld until symptoms return to Grade 2 or below. | Agree (60)  Disagree (10) |  | In my clinical experience, there are circumstances in which the management of brentuximab vedotin dosage for patients with Grade 3 sensory peripheral neuropathy, on a regimen of brentuximab vedotin (1.8 mg/kg) with cyclophosphamide, doxorubicin and prednisone (CHP), may differ from the approach described in the SmPC | Agree (58)  Disagree (33) |
| For patients with Grade 3 motor peripheral neuropathy on a regimen of brentuximab vedotin (1.8 mg/kg) with cyclophosphamide, doxorubicin and prednisone (CHP), as used for peripheral T-cell lymphoma (ECHELON-2 trial), brentuximab vedotin treatment should be discontinued. | Agree (67)  Disagree (8) |  | For patients with Grade 3 motor peripheral neuropathy on a regimen of brentuximab vedotin (1.8 mg/kg) with cyclophosphamide, doxorubicin and prednisone (CHP), as used for peripheral T-cell lymphoma (ECHELON-2 trial), brentuximab vedotin dose should be discontinued or withheld until symptoms reduce to Grade 2 or below. | **Agree (70)**  Disagree (0) |  | | |
| For patients with Grade 2 peripheral neuropathy on a regimen of brentuximab vedotin (1.2 mg/kg) with doxorubicin, vinblastine and dacarbazine (AVD), as used for previously untreated Hodgkin lymphoma (ECHELON-1 trial), brentuximab vedotin dose should be reduced to 0.9 mg/kg up to a maximum of 90 mg every 2 weeks. | Agree (60)  Disagree (10) |  | For patients with Grade 2 and above peripheral neuropathy on a regimen of brentuximab vedotin (1.2 mg/kg) with doxorubicin, vinblastine and dacarbazine (AVD), as used for previously untreated Hodgkin lymphoma (ECHELON-1 trial), vinblastine dose modification may be considered before adjusting the dose of brentuximab vedotin. | **Agree (78)**  Disagree (0) |  | | |
| For patients with Grade 3 peripheral neuropathy on a regimen of brentuximab vedotin (1.2 mg/kg) with doxorubicin, vinblastine and dacarbazine (AVD), as used for Hodgkin lymphoma (ECHELON-1 trial), brentuximab vedotin dose should be held until improvement to Grade 2 or lower, then restarted at 0.9 mg/kg up to a maximum of 90 mg every 2 weeks. | **Agree (80)**  Disagree (0) |  |  |  |  |  |  |
| For patients with Grade 4 peripheral neuropathy, brentuximab vedotin treatment should be discontinued. | **Agree (92)**  Disagree (0) |  | | | | | |
| Patient choice is a primary consideration when clinical decisions are made regarding brentuximab vedotin dose modifications in response to peripheral neuropathy. | Agree (62)  Disagree (15) |  | Clinical decisions regarding brentuximab vedotin dose modifications in response to peripheral neuropathy should be based on a combination of patient choice and clinician expertise. | **Agree (85)**  Disagree (0) |  | | |
| In your experience, which of the following medications can be effective for pain management in brentuximab vedotin-induced peripheral neuropathy? | Duloxetine (46)  Pregabalin (69)  Gabapentin (69)  Topical treatments (e.g. lidocaine) (23)  Other [please specify] (31) |  | Pain medications such as duloxetine, pregabalin and gabapentin may be considered for pain management in brentuximab vedotin-induced peripheral neuropathy, though the effectiveness of these agents is variable. | **Agree (92)**  Disagree (0) |  | | |
| Pain medications (e.g. duloxetine, pregabalin and gabapentin) prescribed while patients are still undergoing brentuximab vedotin treatment introduce a risk of masking peripheral neuropathy symptoms. | Agree (0)  Disagree (42) | ***Deprioritised*** | | | | | |
| Pain medications may be considered once the patient has established peripheral neuropathy. | Agree (38)  Disagree (38) | ***Deprioritised*** | | | | | |
| Immunosuppressive therapies (e.g. corticosteroids, intravenous immunoglobulin) may be considered for Grade 3/4 brentuximab vedotin-induced peripheral neuropathy, depending on clinical and neurophysiological presentation (e.g. for patients with brentuximab vedotin-induced Guillain-Barre Syndrome). | Agree (45)  Disagree (9) |  | Immunosuppressive therapies (e.g. corticosteroids, intravenous immunoglobulin) may be considered for patients with brentuximab vedotin-induced Guillain-Barre Syndrome, confirmed by a nerve conduction study.^*^ | **Agree (80)**  Disagree (10) |  | Intravenous immunoglobulin or plasma exchange may be considered for patients diagnosed with brentuximab vedotin-induced Guillain-Barre Syndrome, confirmed by a nerve conduction study.^*^ | **Agree (100)**  Disagree (0) |
| Given their potential to exacerbate neuropathy, patients undergoing treatment with brentuximab vedotin should be carefully monitored to avoid excessive intake of supplements containing pyridoxine (vitamin B6). | **Agree (73)**  Disagree (18) |  | | | | | |
| Patient choice is an important aspect of pharmaceutical management of brentuximab vedotin-induced peripheral neuropathy. Clinicians should discuss the available management options with patients during appointments to facilitate shared decision-making. | **Agree (100)**  Disagree (0) |  | | | | | |
| In your experience, patient choice and lifestyle are primary considerations in deciding whether to prescribe pain management medications for brentuximab-induced peripheral neuropathy. | **Agree (92)**  Disagree (8) |  | | | | | |

Consensus was pre-defined as ≥70% panellists agreeing/disagreeing with a given statement. For multiple choice questions, consensus was reached where ≥70% panellists selected an option. Where panellists answered “not applicable”, their response was removed from the consensus calculation. **Abbreviations**: AVD: doxorubicin, vinblastine and dacarbazine; BV: brentuximab vedotin; CHP: cyclophosphamide, doxorubicin and prednisone; PN: peripheral neuropathy; SmPC: Summary of Product Characteristics. ^*^While these statements stemmed from the Round 1 statement relating to pharmaceutical management, they were grouped under “diagnostic processes and considerations” for Rounds 2 and 3, as they partially related to NCSs.

Supplementary Table 5. Flowchart of statements on the non-pharmaceutical management of BV-induced PN.

| Round 1 | Outcome (% of panellists) |  | Round 2 | Outcome (% of panellists) |
| --- | --- | --- | --- | --- |
| Non-pharmaceutical interventions should be integrated into treatment plans for brentuximab vedotin-induced peripheral neuropathy as standard practice, to be guided by a multidisciplinary team including specialists beyond neurologists, haematologists, dermatologists and nurses. | **Agree (92)**  Disagree (0) |  | | |
| In your experience, please select the non-pharmaceutical interventions that you have found to be effective for alleviating symptoms of brentuximab vedotin-induced peripheral neuropathy. | Acupuncture (46)  Vitamin B supplementation (15)  Cannabinoids (23)  **Physical therapy (77)**  Oncology massage (38)  Cognitive behavioural therapy (23)  Cryotherapy (8)  Transcutaneous electrical nerve stimulation (TENS) (23)  Other [please specify] (23) |  | | |
| There is a need for more research on the efficacy of non-pharmaceutical interventions for brentuximab vedotin-induced peripheral neuropathy. | **Agree (92)**  Disagree (0) |  | | |
| Physical and occupational therapists are typically responsible for administering non-pharmaceutical interventions for brentuximab vedotin-induced peripheral neuropathy, and these interventions may be supplemented by psychological support from psychologists and counsellors. | Agree (69)  Disagree (15) |  | Physical and occupational therapists can provide non-pharmaceutical interventions to improve the physical quality of life in patients with brentuximab vedotin-induced peripheral neuropathy. | **Agree (100)**  Disagree (0) |
|  |  |  | Psychologists and counsellors can provide non-pharmaceutical support to address the psychological impact of brentuximab vedotin-induced peripheral neuropathy on patients’ quality of life. | **Agree (77)**  Disagree (15) |

Consensus was pre-defined as ≥70% panellists agreeing/disagreeing with a given statement. For multiple choice questions, consensus was reached where ≥70% panellists selected an option. Where panellists answered “not applicable”, their response was removed from the consensus calculation. **Abbreviations**: BV: brentuximab vedotin; PN: peripheral neuropathy.

Supplementary Table 6. Flowchart of statements on future directions and research priorities.

| Round 1 | Outcome (% of panellists) |  | Round 2 | Outcome (% of panellists) |
| --- | --- | --- | --- | --- |
| In your experience, please select from the following list the aspects of brentuximab vedotin-induced peripheral neuropathy where there is a need for clear guidelines. | **Diagnosis (77)**  Dose modification (69)  Dose delay (62)  **Pharmaceutical management (85)**  **Non-pharmaceutical management (85)**  Other [please specify] (23) |  | | |
| Further research is needed regarding the predictive factors of severe, irreversible peripheral neuropathy. | **Agree (92)**  Disagree (0) |  | | |
| More focus is needed on patient centricity when considering decisions around the management of brentuximab vedotin-induced peripheral neuropathy. To address this, there is a need to leverage patient and public involvement and engagement (PPIE) to ensure that patient needs are being addressed. | **Agree (77)**  Disagree (8) |  | | |

Consensus was pre-defined as ≥70% panellists agreeing/disagreeing with a given statement. For multiple choice questions, consensus was reached where ≥70% panellists selected an option. Where panellists answered “not applicable”, their response was removed from the consensus calculation. **Abbreviations**: BV: brentuximab vedotin; PN: peripheral neuropathy; PPIE: patient and public involvement and engagement.
